# Foreign Body Imbedded in the Cheek for Eight Months

**Published:** 1899-07

**Authors:** Charles S. Briggs

**Affiliations:** Nashville, Tenn.


### Foreign Body Imbedded in the Cheek for Eight Months.

BY CHARLES S. BRIGGS, A.M., M.D., OF NASHVILLE, TENN.

Reported at a meeting of Medico-Chirurgical Club, April 28, 1899.

Katie M., æt. nine, resident of North Nashville, while walking in the street, was run against by a boy and knocked down, striking forcibly on the sidewalk upon her face receiving a lacerated wound on the left side of the face above the upper lip. The wound bled rather profusely, but was checked by cold-water applications and pressure. The edges were approximated by adhesive plaster. The parts inflamed considerably, necessitating the removal of the plaster strips and the application of poultices, which were applied under the direction of her family physician. The inflammation gradually subsided, but the tissues over the entire side presented an eruption of angry appearance.

When brought to me for examination, there was found in addition to the eruption above mentioned two small spots, one near the median line, the other considerably smaller, near the parotid region. These points were covered by thin, livid skin, and pressure upon one caused an elevation in the other. Palpation discovered an unnatural induration extending from one point to the other for two inches, the line of demarcation being horizontal and almost in the line of Steno's duct.

An ointment was prescribed to reduce the inflammation and an appointment was made for the patient to go to the Infirmary and have the parts opened under chloroform. A guarded diagnosis was made of injury to Steno's duct with salivary calculus. Sunday, March 26th, under chloroform anesthesia, both soft places were incised, evacuating a thin fluid resembling saliva. A probe introduced into the wound came into contact with a hard substance. A pair of forceps injected into the larger incision grasped the foreign body and it was readily withdrawn. It proved to be a cedar splinter which I have on exhibit to the fellows of the Club. The interesting feature of this case is that a foreign body of the dimensions of this splinter could remain so long in the delicate tissues of the cheek and cause so little local disturbance of the parts.

